# Platinum-Based Chemotherapy Secures Effective Local Control for Very Advanced Hereditary Triple-Negative Breast Cancer with Life-Threatening Bleeding

**DOI:** 10.7759/cureus.1455

**Published:** 2017-07-10

**Authors:** Arvids Irmejs, Peteris Loza, Elina Skuja, Janis Gardovskis

**Affiliations:** 1 Institute of Oncology, Continuing Education, Riga Stradins University, Latvia; 2 Breast Unit, Riga Stradins University Hospital, Riga Stradins University, Latvia; 3 Chief of Surgical Department, Riga Stradins University Hospital, Riga Stradins University, Latvia

**Keywords:** breast cancer, platinum-based chemotherapy, response to chemotherapy, chemotherapy, neoadjuvant chemotherapy, triple negative breast cancer, hereditary breast cancer, bleeding breast cancer, breast cancer local control

## Abstract

Here we report the case of a noncompliant 50-year-old female patient with high-grade, triple-negative breast cancer (TNBC) and strong family cancer history. She only agreed to start treatment after being admitted to the hospital with advanced stage disease and severe anaemia resulting from bulky, ulcerated, and actively bleeding tumor. Therapy was promptly started with platinum-based chemotherapy, resulting in extremely rapid clinical remission and complete control of local symptoms. In conclusion, we hypothesise that even a single course of platinum-based chemotherapy could bring under control life-threatening complications in hereditary TNBC and, therefore, it should be administered without hesitation in emergency circumstances if the patient can tolerate one dose of the medication.

## Introduction

According to the World Health Organization (WHO), breast cancer is the most common cancer in women worldwide. It is estimated that more than 1.7 million new cases and 0.5 million breast cancer deaths occurred among women worldwide in 2012. In developed countries,  due to population-based screening programs, the majority of cases are diagnosed in the early stage [1]. However, a significant proportion of patients present with advanced stage disease where treatment options are limited and achieving satisfactory results can be challenging for clinicians.

This case report is about a 50-year-old female patient with hereditary triple-negative breast cancer (TNBC) who did not comply with initial chemotherapy and, six months later, reappeared in the emergency room with severe anaemia resulting from a bulky, ulcerated, and actively bleeding tumor.  

## Case presentation

This 50-year-old patient initially presented in August 2016 complaining of a lump in her right breast for approximately one year. Family history included ovarian cancer (sister, diagnosed at age 52) and breast cancer (sister of father, diagnosed at age 65). She had been postmenopausal since age 49 and her previous mammogram in August 2015 did not indicate any abnormalities.

At the time of initial presentation, the cancer in her right breast was already locally advanced. Ultrasound examination of right axilla showed at least three specific lymph nodes. Fine needle aspiration (FNA) from suspicious contralateral left-sided axillary lymph node was negative. Core biopsy confirmed infiltrative ductal carcinoma, Grade 3 with focal necrosis, estrogen receptor (ER) negative, progesterone receptor (PR) negative, and human epidermal growth factor receptor 2 (HER2) negative. Genetic testing for three locally common breast cancer susceptibility gene 1 (BRCA1) mutations (*4153delA, C61G, 538insC*) was negative. The patient was recommended to undergo complete BRCA1/2 gene testing, but so far has not performed it. One potential reason is that complete BRCA1/2 gene testing in Latvia has no public funding and the patient herself has to cover all costs. However, the patient has a strong family cancer history matching the diagnostic criteria for hereditary breast ovarian syndrome. The probability of finding BRCA1/2 gene mutation is reasonably high, and regardless of genetic test results, this should be considered as a hereditary breast cancer [2]. 

The decision was to commence treatment with neoadjuvant chemotherapy; however, the patient failed to attend the oncology clinic for her scheduled treatments. 

**Figure 1:**
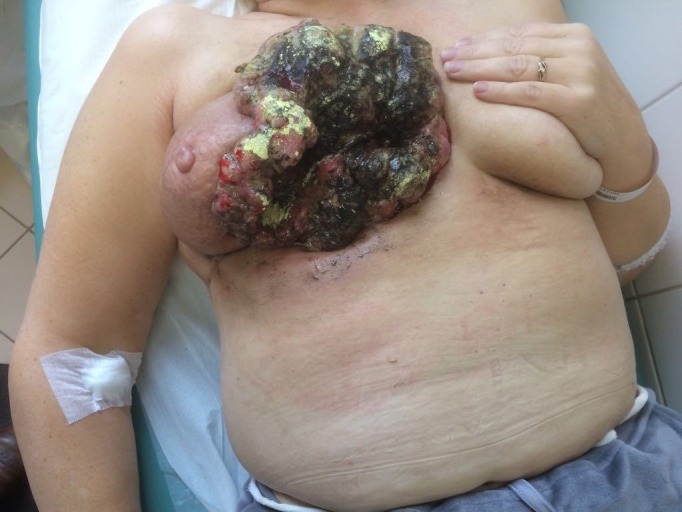
Picture of tumor before treatment

Six months later, in February 2017, she presented in the emergency room with symptoms of severe anaemia  (blood haemoglobin 3,7 g/dL),  Physical examination revealed bulky (7 X 15 cm), ulcerated, and actively bleeding carcinoma, eroding most of medial part of the right breast. The lesion was fixed to the chest wall and was producing an offensive smell (Figure 1).

Apart from carcinomatous intoxication, her general condition was relatively stable and hemodynamics were stable. She was administered eight units of blood and four units of fresh frozen plasma (FFP). Bleeding was temporarily controlled by a pressure bandage.

**Figure 2:**
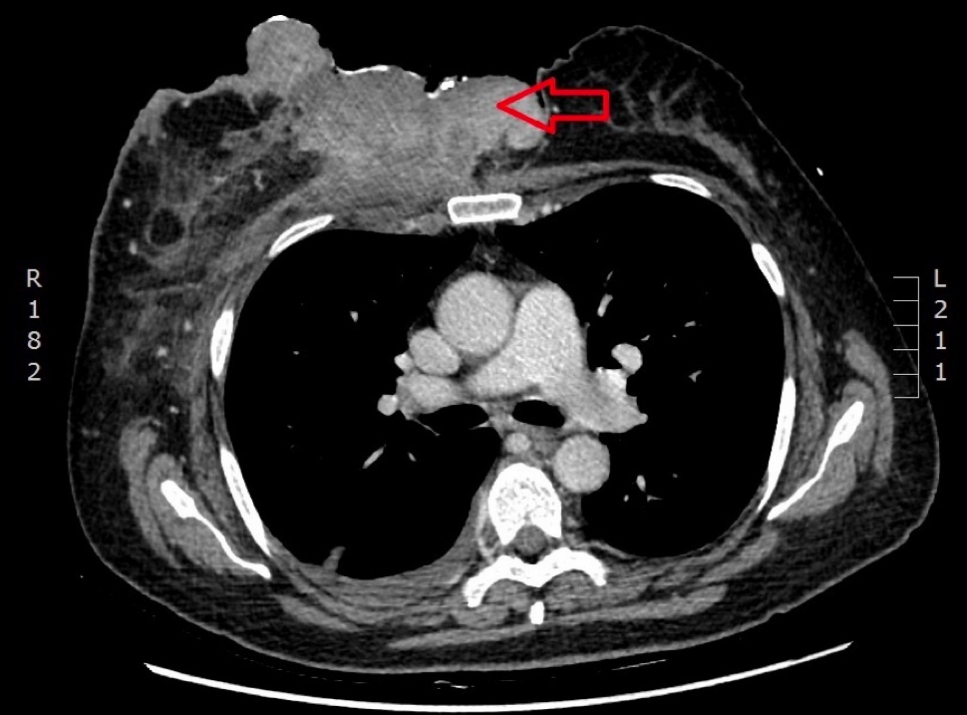
CT scan before treatment Large tumor mass fixed to the chest wall (*arrow*)

A chest computed tomography (CT) scan in February 2017 showed a large infiltrative tumor mass adherent to chest wall muscles and the cartilage of the fourth rib and sternum (Figure 2). The right internal mammary vessels were involved. Mediastinal lymphadenopathy with multiple lymph nodes up to 1 cm in diameter were detected. Moreover, multiple (at least five) specific right axillary nodes up to 1.5 cm in diameter were present. A small amount of pleural effusion on the right side was diagnosed. A bone scan showed no abnormality. The blinical stage was T4cN3bMx (TNM classification for staging of breast cancer according to NCCN guideline V 1.2016).  As no bone, lung, and liver metastasis were confirmed, we cannot completely rule out that mediastinal lymph nodes were unspecific; hence, M stage is questionable.

Tumor marker carcinoma antigen 125 (CA-125) was elevated (49.7 U/mL); however, markers CA 15-3 and carcinoembryonic antigen (CEA) were within normal range.

Because of repeated severe bleeding episodes during change of dressings and the offensive smell, which extended ouside the ptient's room, immediate measures were indicated for life saving and hygienic purposes. The general surgeons in charge considered surgical removal, but it was rejected by the breast surgeon because surgical intervention would result in an extensive chest wall defect which would then require autologous flap reconstruction. This caries high risk of severe complications and should be considered only after failure of systemic therapy. Moreover, the patient’s general condition was relatively poor and M stage was inconclusive. The option of arterial embolisation was proposed by the breast surgeon, but it was rejected by the invasive radiologist, because at that particular moment there was no active arterial bleeding. 

Ten days after admission to the hospital, neoadjuvant chemotherapy with three-week intervals of doxorubicin 60 mg/m^2^ and carboplatin in dose AUC-5 (area under the plasma concentration time curve) was commenced. 

**Figure 3:**
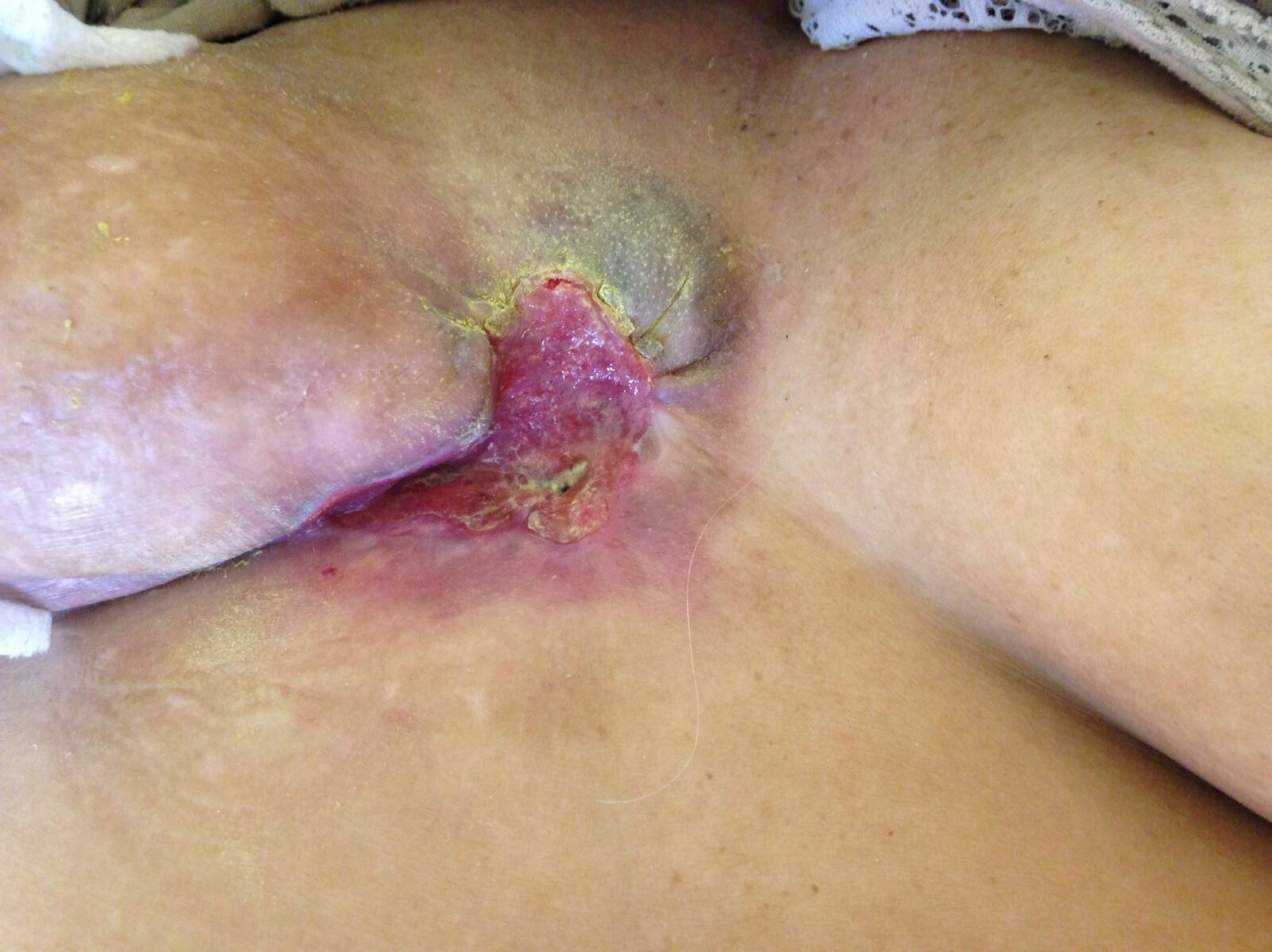
Picture of tumor after two cycles of chemotherapy

After just two cycles of chemotherapy, there was unexpectedly rapid clinical response and effective control of local symptoms (Figure 3). The tumor gradually shrunk to the centre with healthy skin forming from the periphery. 

**Figure 4:**
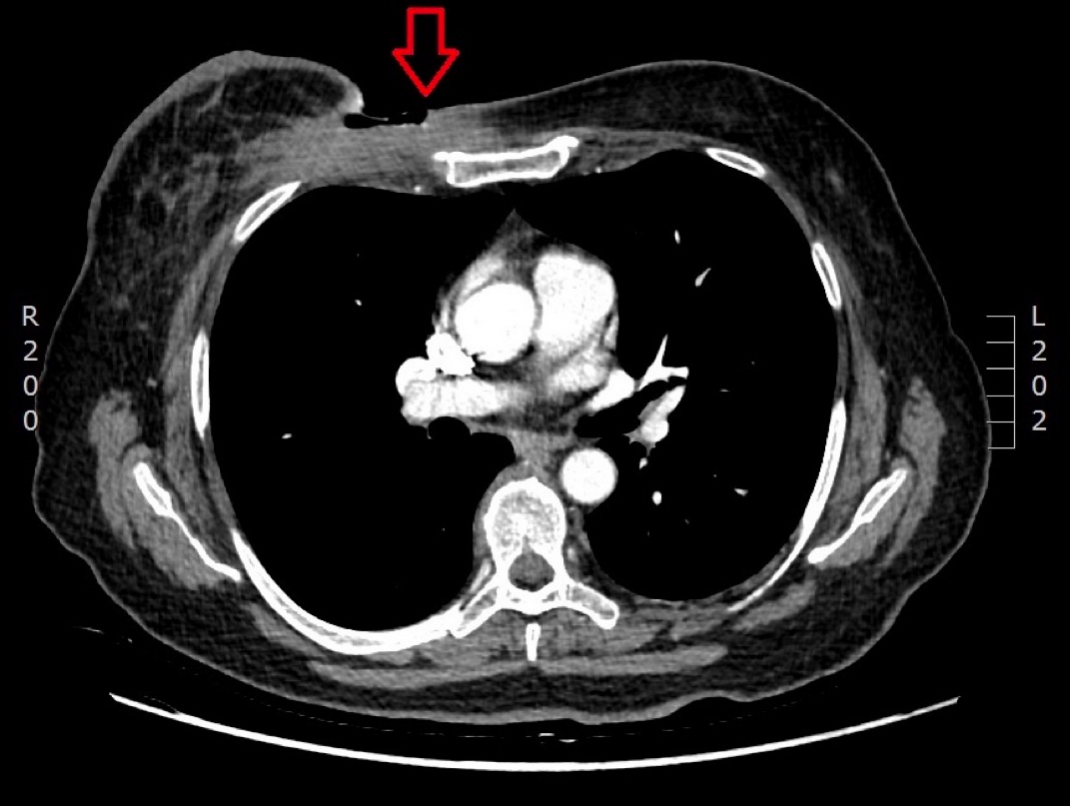
CT scan after two cycles of chemotherapy The primary bulky tumor has significantly reduced in size (*arrow) *

Repeated CT chest/abdomen scans in April 2017 indicated marked remission of the primary tumour, complete remission of mediastinal lymphadenopathy, and no radiologic evidence of any other distant metastasis (Figure 4). However, specific right axillary lymphadenopathy remained present.

We are currently eager, waiting for further loco-regional remission as the patient continues with chemotherapy. Afterwards, surgery and RT will be considered for further local control.

## Discussion

From a historical perspective, surgery has been the method of choice for local control (including hygienic purposes) of advanced breast cancer. Over the last decades, increasing evidence has emerged on the role of systemic therapy for the local control of the disease. The role of chemotherapy and endocrine therapy in local control of advanced breast cancer is well documented [3]. However, the use of platinum-based therapies for treatment of TNBC in patients with strong family cancer history and/or BRCA1/2 mutations is a relatively recent development. There are several reports on the effectiveness of cisplatinum use in the metastatic and neoadjuvant setting for BRCA1 positive breast cancer that are consistent with our report [4-6]. Also, recent evidence from systematic reviews and meta-analysis of randomised-controlled trials suggest the benefits of adding platinum to conventional anthracycline-based chemotherapy in patients with locally advanced and metastatic TNBC [7].  In case of TNBC, platinum-based therapy is associated with significantly higher complete pathologic response rates, which is believed to be an independent indicator of response to treatment as well as overall prognosis [8]. However, further research is mandatory to support these findings.

Authors Ishizuna K, et al. [9] have reported two cases of locally advanced TNBC where bleeding from tumor and local control was achieved with chemotherapy plus targeted agents (weekly paclitaxel plus bevacizumab). 

To the best of our knowledge, this is the first report in the literature on the use of platinum-based chemotherapy for control of life threatening bleeding in case of T4c(TNM classification for staging of breast cancer according to NCCN guideline V 1.2016) advanced hereditary triple-negative breast cancer.

In our case, the interval between admission to hospital and initiation of chemotherapy was 10 days. This is a relatively short period of time; however, in view of the significant and disturbing symptoms which affected not only the patient herself but also other patients and hospital staff,  retrospectively we admit that this period could have been even shorter.  

## Conclusions

Our case of exceptional responder raises several hypothesis that should be confirmed by a larger series: 1) platinum-based therapy could be effective for very advanced TNBC with strong family cancer history and/or BRCA1/2 mutation; 2) even a single course of chemotherapy could bring under control life-threatening complications and therefore should be administered without hesitation in those circumstances, providing the patient can tolerate one dose of the medication.
